# Helminth Elimination in the Pursuit of Sustainable Development Goals: A "Worm Index" for Human Development

**DOI:** 10.1371/journal.pntd.0003618

**Published:** 2015-04-30

**Authors:** Peter J. Hotez, Jennifer R. Herricks

**Affiliations:** 1 Department of Pediatrics and Molecular Virology and Microbiology, National School of Tropical Medicine, Baylor College of Medicine, Houston, Texas, United States of America; 2 Sabin Vaccine Institute and Texas Children’s Hospital Center for Vaccine Development, Houston, Texas, United States of America; 3 James A. Baker III Institute for Public Policy, Rice University, Houston, Texas, United States of America; 4 Department of Biology, Baylor University, Waco, Texas, United States of America; Lindsley F. Kimball Research Institute, UNITED STATES

A new “worm index” confirms a strong association between helminth infections and impaired human development. In June 2012, a landmark United Nations conference on sustainable development was held in Rio de Janeiro, Brazil. Known as Rio+20, the conference focused on a new set of sustainable development goals (SDGs) that would begin following the sunset of the Millennium Development Goals (MDGs) in 2015 [1]. Like the MDGs, the SDGs will focus on poverty reduction, gender equality, and human and economic development, but in addition the SDGs will also emphasize food security and key issues related to the environment, such as climate change, the oceans, and biodiversity [1].

To date, unlike MDG 6, which includes combatting “AIDS, malaria, and other diseases,” the preliminary SDGs outlined at Rio+20 do not list specific diseases. Yet over the last decade, increasing evidence links the major neglected tropical diseases (NTDs) to a significant adverse impact on both human and economic development, especially for the major helminth infections, i.e., hookworm and the intestinal helminth infections, schistosomiasis, and lymphatic filariasis [2]. According to the Global Burden of Disease Study 2010 (GBD 2010), these helminth infections rank among the leading NTDs in terms of disability-adjusted life years (DALYs), accounting for approximately 10 million DALYs [3].

Helminth infections exert major effects on the essential components that comprise human development indices. Established by the economists Amartya Sen and Mahbub ul Haq and their colleagues in the 1990s [4], the human development index (HDI) is a summary of measure of the major dimensions of human development, including standard of living, educational attainment and years of schooling, and years of life lived with good health [5]. There is evidence linking lymphedema from lymphatic filariasis to diminished labor and worker productivity, as well as excessive health care burdens especially in rural areas [2,6–8]. In addition, data emerging over the last two decades indicate that intestinal helminth infections and schistosomiasis diminish or reduce childhood nutrition, development, cognition, and education [6,9–15].

With regards to educational effects, Miguel and Kremer found that in Kenya, school-based deworming (for intestinal helminths and schistosomes) led to a 7.5 percentage point average gain in primary school participation and a reduction in school absenteeism by at least 25% [9]. Moreover, persistent hookworm infections in childhood in early 20th century America were shown to depress eventual educational attainment by more than two years, and future wage earning by 40% [8], while in Kenya, adults who received deworming treatment as children were also found to be more productive in terms of average working hours and wage-earning [13]. However, the extent to which deworming improves education and other aspects of human development has been questioned and is undergoing re-evaluation [11,12,14,15]. Finally, there are potent interactions between the helminth infections highlighted above and malaria or HIV/AIDS [16], which may further affect human development.

The United Nations Development Programme (UNDP) published the most recent human development indices for 187 nations in 2014 [17]. Not surprisingly, countries such as Norway and the United States exhibited the highest HDI, approaching the 1.000—the maximum HDI possible, while Democratic Republic of Congo (DR Congo) and Niger exhibited the lowest HDI, with values just over 0.330 [17]. In between were countries such as the large middle-income BRICS nations—Brazil, Russia, India, China, and South Africa—which ranged between 0.500 and 0.800 [17], as shown in this map of the world’s nations according to their HDIs: http://en.wikipedia.org/wiki/Human_Development_Index.

**Fig 1:**
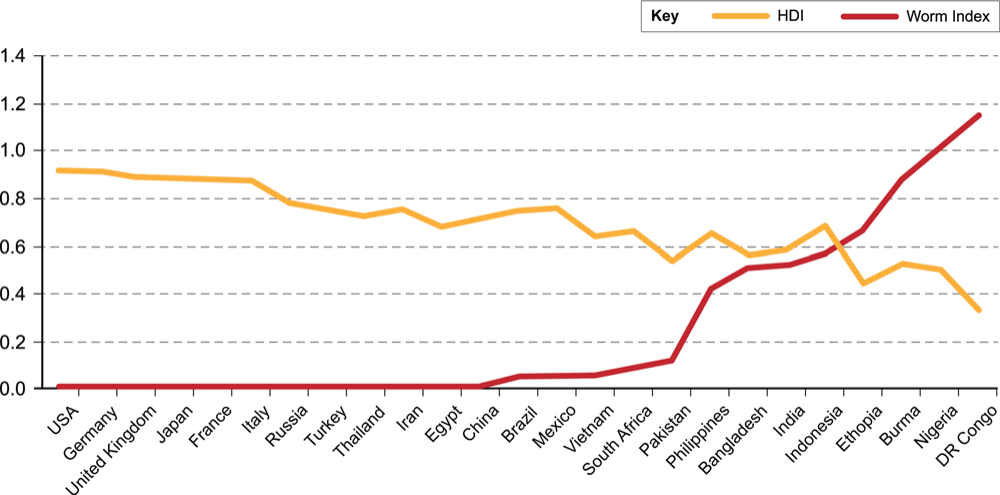
Comparison of worm index vs. HDI (based on data in Table 1).

As shown in Table 1 and Fig 1, we recently compared, the 2014 UNDP HDIs to the three highest disease burden (as determined by their DALYs [3]) helminth infections—intestinal helminth infections, schistosomiasis, and lymphatic filariasis—in the 25 most populated nations. Together these nations comprise more than three-quarters of the world’s population [18]. In order to assign a single value to these important helminth infections we calculated a “worm index” based on recent World Health Organization (WHO) data for:

1. the number of school-aged children requiring annual deworming for their intestinal helminth infections [19],
2. the populations requiring preventive chemotherapy for lymphatic filariasis [20], and
3. the number of school-aged children requiring preventive chemotherapy treatments for schistosomiasis [21].

**Table 1:** The worm index and HDI in the world’s 25 most populated nations.

**Country**	**Population [18]**	**STH** ^a^ **[19] 2012**	**LF [20] 2013**	**Schisto** ^a^ **[21] 2013**	**Total Worms** ^b^	**Worm Index** ^c^	**Worm Index rank**	**HDI [5, 17]**	**HDI Rank [5,17]**
**China**	1.393 billion	18.4 million	0	0.1 million	18.5 million	**0.013**	14	**0.719**	91
**India**	1.267 billion	170.7 million	489.1 million	0	659.8 million	**0.521**	6	**0.586**	135
**USA**	323 million	0	0	0	0	**0**	16	**0.914**	1
**Indonesia**	253 million	43.6 million	99.7 million	<0.1 million	143.3 million	**0.566**	5	**0.684**	108
**Brazil**	202 million	8.9 million	0.3 million	1.5 million	10.7 million	**0.052**	13	**0.744**	79
**Pakistan**	185 million	21.8 million	0	0	21.8 million	**0.117**	9	**0.537**	146
**Nigeria**	179 million	43.6 million	114.3 million	23.2 million	181.1 million	**1.012**	2	**0.504**	152
**Bangladesh**	159 million	31.4 million	49.7 million	0	81.1 million	**0.510**	7	**0.558**	142
**Russia**	142 million	0	0	0	0	**0**	16	**0.778**	57
**Japan**	127 million	0	0	0	0	**0**	16	**0.890**	15
**Mexico**	124 million	7.0 million	0	0	7.0 million	**0.056**	11	**0.756**	71
**Philippines**	100 million	22.2 million	19.5 million	0.5 million	42.2 million	**0.422**	8	**0.660**	117
**Ethiopia**	97 million	22.8 million	30 million	12.0 million	64.8 million	**0.668**	4	**0.435**	173
**Vietnam**	93 million	5.2 million	0	0	5.2 million	**0.056**	12	**0.638**	121
**Egypt**	84 million	0	0.6 million	0.1 million	0.7 million	**0.008**	15	**0.682**	110
**Germany**	83 million	0	0	0	0	**0**	16	**0.911**	2
**Iran**	78 million	0	0	0	0	**0**	16	**0.749**	75
**Turkey**	76 million	0	0	0	0	**0**	16	**0.759**	69
**DR Congo**	69 million	19.6 million	49.1 million	10.2 million	78.9 million	**1.143**	1	**0.338**	186
**Thailand**	67 million	0	0	0	0	**0**	16	**0.722**	89
**France**	65 million	0	0	0	0	**0**	16	**0.884**	20
**United Kingdom**	63 million	0	0	0	0	**0**	16	**0.892**	3
**Italy**	61 million	0	0	0	0	**0**	16	**0.872**	26
**Burma**	54 million	8.1 million	39.5 million	0	47.6 million	**0.881**	3	**0.524**	150
**South Africa**	53 million	2.3 million	0	2.5 million	4.8 million	**0.090**	10	**0.658**	118
**Total**	5.4 billion				1.37 billion	**0.254**		**0.702**	

^a^ Data for school-aged children only

^b^ Calculated by adding columns 3–5

^c^ Calculated by dividing number of total worm infections obtained in column 6 by population in column 2

These three values were added for each of the 25 most populated nations and then divided by their 2014 population estimate. The resulting worm indices varied between 0 and a value just above 1.0.

At greater than 1.0 DR, Congo and Nigeria each exhibited the highest worm indices, followed by Burma with a worm index that exceeded 0.8, and Ethiopia whose worm index exceeded 0.6. The nations of Bangladesh, India, Indonesia, and Philippines had worm indices in the 0.4 to 0.6 range, while Brazil, China, Egypt, Pakistan, Mexico, South Africa, and Vietnam were less than 0.4 but still above zero. The worm indices for France, Germany, Iran, Italy, Japan, Russia, Thailand, Turkey, United Kingdom, and United States were zero.

As shown in Fig 1, there was a statistically significant negative association (R = −0.8 and *p* < 0.0001) between the worm indices and the HDIs in the world’s most populated countries. The worm index begins to climb as a nation’s HDI falls below 0.750 and it rises precipitously when the HDI drops below 0.600. The countries with the highest worm indices have an HDI less than 0.400.

The exact reasons for the associations highlighted here need to be investigated further. For example, it remains unclear to what extent helminth infections are causes of low human development and what role low human development has in promoting widespread helminthiases. It is possible or even likely that the causes and effects flow in both directions.

Of additional interest was the finding of moderate to high worm indices in India, Indonesia, and Philippines, which the UNDP categorizes as high HDI nations. To better understand this finding, we may look to the concept of “blue marble health,” in which high rates of helminthic and other NTDs are found in pockets of intense poverty in middle- and high-income countries [22,23]. Indeed, some of the populations most affected by NTDs in the world today are poor persons living among the wealthy in a group of 20 (G20) countries [22,23]. Included among these impoverished groups are aboriginal populations [24].

The UNDP 2014 report entitled “Sustaining Human Progress: Reducing Vulnerabilities and Building Resilience” emphasizes vulnerable and excluded populations, including the poor living in wealthier countries and aboriginal groups, while also stressing global commitments to universalism expressed in terms of rights of access to healthcare, education, and other basic services [17].

Given the strong associations between helminthic and other NTDs and mental, physical, and economic human development, vulnerable and excluded populations, and HDI, in the coming months and years it may become essential to give due consideration to eliminating helminth infections as a means to achieve SDGs. Of course, poverty, “Water, Sanitation and Hygiene” (WASH), and nutrition play an important role in health outcomes. As we look to reduce poverty and increase WASH and nutrition, we must also focus on chronic diseases such as helminth infections that reinforce the cycle of poverty and malnutrition. Therefore, we suggest that the NTDs need to be an important consideration in any discussion about the SDGs, and helminth control and elimination as proposed by the 2012 London Declaration for NTDs must be embraced by the SDGs and the sustainable development community [23].

The next steps required to eliminate intestinal helminth infections, schistosomiasis, lymphatic filariasis, and other helminthic NTDs were highlighted in 2011 [25]. They include expanding global efforts to deliver preventive chemotherapy packages of NTD essential medicines for the entire bottom billion at risk for these infections, but in addition, for some helminth infections such as hookworm, schistosomiasis, and onchcerciasis, new tools and strategies will also be required, possibly including anthelminthic vaccines [6,25,26]. Indeed, a survey of almost 400 NTD experts found that mass treatment preventive chemotherapy, while it may be sufficient for eliminating lymphatic filariasis and trachoma, will not be sufficient for eliminating intestinal helminth infections and schistosomiasis [27]. Therefore, we believe that research and development for these new NTD control and elimination strategies and tools will be required, along with expansion of preventive chemotherapy, in order to reduce a nation’s worm index, and should become an essential component of the SDGs [22,23,25,26].
